# Extracellular degradation of a polyurethane oligomer involving outer membrane vesicles and further insights on the degradation of 2,4-diaminotoluene in *Pseudomonas capeferrum* TDA1

**DOI:** 10.1038/s41598-022-06558-0

**Published:** 2022-02-17

**Authors:** Òscar Puiggené, María José Cárdenas Espinosa, Dietmar Schlosser, Stephan Thies, Nico Jehmlich, Uwe Kappelmeyer, Stephan Schreiber, Daniel Wibberg, Joern Kalinowski, Hauke Harms, Hermann J. Heipieper, Christian Eberlein

**Affiliations:** 1 Department of Environmental Biotechnology, Helmholtz Centre for Environmental Research - UFZ, Leipzig, Germany; 2 Department of Environmental Microbiology, Helmholtz Centre for Environmental Research - UFZ, Leipzig, Germany; 3 Institute of Molecular Enzyme Technology, Heinrich Heine University Dusseldorf, Jülich, Germany; 4 Department Molecular Systems Biology, Helmholtz Centre for Environmental Research - UFZ, Leipzig, Germany; 5 Microbial Genomics and Biotechnology, Center for Biotechnology (CeBiTec), Bielefeld, Germany; 6 The Novo Nordisk Foundation Center for Biosustainability, Technical University of Denmark, 2800 Kgs, Lyngby, Denmark

**Keywords:** Microbiology, Environmental microbiology

## Abstract

The continuing reports of plastic pollution in various ecosystems highlight the threat posed by the ever-increasing consumption of synthetic polymers. Therefore, *Pseudomonas capeferrum* TDA1, a strain recently isolated from a plastic dump site, was examined further regarding its ability to degrade polyurethane (PU) compounds. The previously reported degradation pathway for 2,4-toluene diamine, a precursor and degradation intermediate of PU, could be confirmed by RNA-seq in this organism. In addition, different cell fractions of cells grown on a PU oligomer were tested for extracellular hydrolytic activity using a standard assay. Strikingly, purified outer membrane vesicles (OMV) of *P. capeferrum* TDA1 grown on a PU oligomer showed higher esterase activity than cell pellets. Hydrolases in the OMV fraction possibly involved in extracellular PU degradation were identified by mass spectrometry. On this basis, we propose a model for extracellular degradation of polyester-based PUs by *P. capeferrum* TDA1 involving the role of OMVs in synthetic polymer degradation.

## Introduction

For the past 80 years, polyurethane (PU) has been one of the world's most versatile polymers, rising towards a market value expected to surpass 87 billion USD by 2026 worldwide^1^. Chemically, polyurethane is the condensation product of polyisocyanates and polyols, yielding urethane bonds. Nonetheless, polyurethane structure is rather heterogeneous and depends on the plastic monomers used^2^. For instance, ester or ether bonds may be present in the polyol segment^3^. The resulting broad range of materials has applications in many sectors, such as building and construction, furniture production, automotive or medical devices, due to its excellent mechanical properties, stability and enhanced biocompatibility^4^.The high polyurethane demand generates significant amounts of waste globally, of which only 29.7% is recycled, 30.8% is disposed in landfills and 39.5% is recuperated through energy recovery^5,6^. However, each of these methods has several shortcomings including the emergence of toxic by-products (e.g. HCN, NO_x_ and CO), increasing landfill costs and leakage, high performance equipment, as well as environmental and health concerns^7,8^.

In search of sustainable alternatives, biodegradation of PUs has been subject of extensive study^2,9–11^. Due to the presence of urethane bonds in their backbone, polyurethanes are susceptible to hydrolysis by enzymes secreted by microorganisms, thus releasing breakdown products, which may act as a carbon source^12^. Even though fungi (namely *Aspergillus* sp. and *Penicillium* sp.) have been reported as the principal degraders of PU in nature, enzymatic activity has also been associated with different bacterial strains. Among bacteria, some of the well-studied PU-degrading organisms are *Acinetobacter*, *Bacillus subtilis*, *Corynebacterium*, *Comamonas acidovorans*, and members of the genus *Pseudomonas*^3,13^. In the past years, several *Pseudomonas* species have been identified for their degradative potential of various plastic polymers^3,13–16^. Specifically, *Pseudomonas chlororaphis* and *Pseudomonas protegens* (formerly *fluorescens*) Pf-5 and recently the *P. pertucinogena* lineage have been found to degrade polyester-based PUs^14–16^.

Initially, biodegradation often requires microbial attachment on the surface of synthetic polymers. The adherence and colonization of bacteria tend to reduce the resistance of the plastic material, which facilities the accessibility of secreted enzymes to modify the physicochemical properties of the polymers. Inside the complex nature of the biofilms, small and non-replicative spherical nanostructures (20–240 nm) called outer membrane vesicles (OMVs) are constantly released by gram-negative bacteria^17^. Furthermore, some reports show that OMVs may contribute to the biofilm formation^18,19^. OMVs harbor active enzymes and extracellular structures were reported to exhibit catabolic activity in different bacteria (*Pseudomonas*, *Rhodoccocus*, *Amycolatopsis* and *Delftia*) grown on aromatic substrates such as phenanthrene and lignin-rich media^20,21^.

Resilient plastics such as polyethylene (PE) and polystyrene (PS) strongly depend on the formation of a biofilm in order to increase the surface interactions with bacterial cells^22,23^. For example, *Pseudomonas sp*. AK2 showed an enhanced low-density PE degradation through an adapted biofilm compared to planktonic cells^24^. Other strains such as *Bacillus sp*. grown on PS films presented a reduced polymer mass by 23% after 14 days^25^.

Polyester-based PU degrading enzymes in *Pseudomonas* are assumed to be primarily extracellular esterases, lipases and cutinases, which may be membrane-bound or secreted extracellularly^26–28^. These enzymes are involved in a catalytic reaction called hydrolysis, which degrades PU by cleaving the ester bonds. Subsequently, intracellular enzymes and metabolic pathways further mineralize these compounds and use their carbon, nitrogen and energy to grow^29^.

As a result of enzymatic degradation, PU waste in landfills may continuously release environmental pollutants into soil or groundwater, such as 4,4′-methylenedianiline (MDA) and 2,4-toluene diamine (2,4-TDA)^30,31^, which are considered as possible human carcinogen^32^ and pose an environmental risk for species in the aquatic and terrestrial areas^33^. Recently, *Pseudomonas capeferrum* TDA1 was identified as the first bacterial strain capable of degrading 2,4-TDA, as well as a PU oligomer^34^. In that previous report, the enzymes involved in the degradation pathway of 2,4-TDA were proposed and are further confirmed by RNA-seq in this study. Moreover, *Pseudomonas capeferrum* TDA1, its suitability and potential in (bio)technological plastic upcycling has recently acquired significant attention^35,36^.

In order to understand different aspects of the biodegradation process, obtaining reliable gene expression data is vital. Therefore, top notch techniques such as advanced genome annotation, RNA-seq transcriptomics and proteomics were applied to study the pathway of 2,4-TDA degradation as well as adaptive responses to environmental changes^37^ during growth on different carbon sources. Furthermore, the growth on a PU oligomer was examined and extracellular esterase activity was detected in different cell fractions of *P. capeferrum* TDA1. Hence, a model for extracellular degradation of polyurethane via outer membrane vesicles (including outer membrane-bound and periplasmic hydrolases) was suggested in *Pseudomonas capeferrum* TDA1.

## Materials and methods

### Chemicals, media and cultivation conditions

*P. capeferrum* TDA1 was grown in mineral media^38^ containing the following compounds: 7 g Na_2_HPO_4_ × 2 H_2_O; 2.8 g KH_2_PO_4_; 0.5 g NaCl; 0.1 g NH_4_Cl; 0.1 g MgSO_4_ × 7 H_2_O; 10 mg FeSO_4_; 5 mg MnSO_4_; 6.4 mg ZnCl_2_; 1 mg CaCl_2_ × 6 H_2_O; 0.6 mg BaCl_2_; 0.36 mg CoSO_4_ × 7 H_2_O; 0.36 mg CuSO_4_ × 5 H_2_O; 6.5 mg H_3_BO_3_; 10 mg EDTA; 146 μl HCl (37%); per liter of demineralized water. For growth assessement, 3 g/l PU oligomer (Sigma-Aldrich, dihydroxy-functional oligomer, aliphatic urethane of proprietary composition, average M_n_ ~ 320 Dalton) or 2 mM 2,4-TDA or 2 mM 2,4-TDA + succinate were added^34^. Disodium succinate (4 g/l) was used as a control. Growth conditions were implemented as described recently^39^.

### Esterase/lipase assay

Bacterial cells were grown for 1–2 days, harvested in exponential phase and centrifuged. The pellet was washed with 10 mM KNO_3_ and centrifuged once more. Similar to a previous study^40^, supernatants, whole-cell samples and OMVs were assayed for esterase/lipase activity. Briefly, in 96-well plates 20–100 µl of sample were incubated with 200 µM of *p*-nitrophenyl esters (*p*-nitrophenyl butyrate, pivalate, valerate and palmitate were added from 5 mM stocks in ethanol, which were always freshly prepared prior to use) in 100 mM Tris–HCl pH 7.5 buffer (final volume 200 µl). Microplates were incubated at 30 °C for 1 h in Tecan GENios Plus Microplate Reader (Tecan, Männedorf, Switzerland) and measurements at 405 nm proceeded every minute. For activity calculation, slopes were normalized as previously described^41^ by sample volume (i.e. protein amount) and comparison with the negative controls. *p*-Nitrophenyl extinction coefficient ($\varepsilon_{410}$) and optical path length (*d*) were considered constant with values of 11.8 × 10^6^ cm^2^/mol (at pH 7.5) and 0.5925 cm, respectively.

The normalization equation employed was the following:

$$A = \frac{{\Delta E_{405} }}{{\varepsilon_{405} \cdot d \cdot \frac{{V_{sample} }}{{V_{total} }}}}$$

where *A* refers to activity in mol L^-1^ min^-1^ and $\Delta E_{405}$ refers to the absorbance increase per min at 405 nm. The molar extinction coefficient ($\varepsilon_{405} )$ for *p-*nitrophenol at pH 7.5 and 410 nm was obtained from Kademi et al*.* (2000)^41^ and was assumed to be equal at 405 nm.

### Outer membrane vesicles (OMVs) isolation

OMV were isolated from *P. capeferrum* TDA1 samples grown on PU oligomer (Figure S1) or succinate in exponential growth phase. Samples were grown until an OD_560_ 0.4–0.5. Cells were then harvested, and supernatant was filtered through a 0.45-µm pore size membrane (Sartorius AG, Göttingen, Germany). Then, isolation was carried out as detailed in Kadurugamuwa and Beveridge (1995)^42^. OMVs were harvested through ultracentrifugation at 100,000 g for 3 h at 4 °C (L-90 K, Rotor-Type 50.2 Ti, Beckmann, USA). Supernatant was then discarded and OMV pellet was resuspended with the remaining supernatant. Protein concentration was also obtained by Bradford measurement (BioRad). Relative values of OMV release were obtained as previously reported^43^ by comparing absolute OMV concentration after isolation to total bacterial protein.

### Liquid chromatography-mass spectroscopy (LC–MS)

OMVs from cultures with PU oligomer and succinate were harvested and processed by LC–MS/MS analysis. Samples were directly treated with trypsin. Peptide lysates were re-dissolved in water containing 0.1% formic acid (20 µL) and analyzed on a Q Exactive HF instrument (Thermo Fisher Scientific, Waltham, MA, USA) equipped with a TriVersa NanoMate source (Advion, Ithaca, NY, USA) in LC chip coupling mode.

Peptide lysates were injected on a trapping column (Acclaim PepMap 100 C18, 3 μm, nanoViper, 75 μm × 2 cm, Thermo Fisher Scientific) with 5 μL/min by using 98% water/2% ACN 0.5% trifluoroacetic acid, and separated on an analytical column (Acclaim PepMap 100 C18, 3 μm, nanoViper, 75 μm × 25 cm, Thermo Fisher Scientific) with a flow rate of 300 nL/min over 80 min. Mobile phase was 0.1% formic acid in water (A) and 80% ACN/0.08% formic acid in water (B). Raw LC–MS/MS data were processed with Proteome Discoverer (v2.4, Thermo Fisher Scientific). Search settings for the Sequest HT search engine were set to trypsin (Full), max. missed cleavage: 2, precursor mass tolerance: 10 ppm, fragment mass tolerance: 0.02 Da. The LC raw files were searched against the protein-coding sequences of *Pseudomonas* sp. TDA1 (Uniprot Proteome ID UP000476571). The false discovery rates (FDR) were determined with the node Percolator embedded in Proteome Discoverer and was set for the protein FDR (< 1%). Subcellular location of resulting proteins, if not already characterized, was predicted via CELLO v2.5^44^.The mass spectrometry proteomics data have been deposited to the ProteomeXchange Consortium via the PRIDE^45^ partner repository with the dataset identifier PXD029164.

### RNA extraction and quantification

Exponentially growing bacterial cells were mixed with in RNA Later solution and centrifuged (5 min at 20,000 g) to collect cells and to discard the supernatant. Total RNA from samples containing succinate or succinate + 2,4-TDA as a carbon source was extracted using the RNeasy kit (Qiagen, Düsseldorf, Germany) according to the manufacturer’s protocol including some modifications: First, the cell solution was centrifuged (5 min at 20,000 g) and the supernatant removed. Each sample was mixed with the buffer RLT (700 µl) + β-mercaptoethanol (7 µl), stored on ice for 2 min and transferred to lysing matrix B tubes for homogenization using FastPrep-24 (MP Biomedicals, Inc) during 35 s at 6.5 m/s. Then, the supernatant was removed to a new 1.5-ml low binding micro-centrifuge tube, an equal volume of ethanol (70%) was added, and the samples were centrifuged for 30 s at 8,000 g. To remove DNA contamination, DNA-free DNA removal kit (Thermo- Scientific, Waltham, United States) was added to each sample and the mixture incubated for 1 h at 37 °C.

For cells grown on 2,4 TDA, a modified RNA extraction protocol was applied to account for the presence of polyphenols, polysaccharides and secondary metabolites which interfere with or degrade the RNA^42^. After centrifugation, 0.5 ml of RNAzol® RT (Sigma-Aldrich, St. Louis, USA) were added to the pellets and re-suspended in the reagent. Each solution was transferred to the lysing matrix B tubes and homogenized. After homogenization, the samples were transferred to 1.5-ml micro-centrifuge tubes and 0.2 ml of RNase-free water were added for DNA, protein, and polysaccharide precipitation according to a protocol published earlier^46^.

Total RNA samples were quantified using a fluorescent RNA-binding dye Qubit Fluorometer (Thermo Fisher, Waltham, United States) according to the manufacturer’s instructions and the RNA integrity was evaluated using an Agilent 2100 Bioanalyzer (Agilent Technologies Inc., Santa Clara, United States) following the manufacturer’s protocols. Samples with integrity numbers (RIN) above 7.0 were selected for further use.

### Ribosomal RNA depletion

Depending on the starting concentration of total RNA, two rRNA depletion methods were used according to the manufacturer’s specifications. A Ribominus Transcriptome Isolation Kit for bacteria (Thermo-Scientific, Waltham, United States) was selected for cells grown on succinate or succinate + 2,4-TDA resulting in total RNA concentrations above 50 ng/µl. The enriched mRNA was then purified and concentrated by ethanol precipitation according to the manual with centrifugation at 15,000 g and precipitation at − 80 °C for 45 min. A riboPOOL Kit (siTOOLs Biotech, Martinsried, Germany) was used exclusively for 2,4-TDA-derived pooled samples with low RNA concentrations (at least 5 ng/µl). After rRNA depletion, all the samples were analyzed using the QuantiFluor kit, a Fluorometer (Promega, Wisconsin, USA) and Agilent 2100 Bioanalyzer.

### RNA-seq library preparation and sequencing

A RNA-seq library was prepared using the NEBNext Ultra II RNA Library Prep Kit for Illumina (New England Biolabs Inc., Massachusetts, USA), according to the manufacturer’s instructions. Briefly, 2.5 ng of total RNA was fragmented using NEBNext First Strand Synthesis Reaction Buffer. After the first and second strand cDNA synthesis, the NEBNext Adaptor was ligated to the cDNA fragments and the enrichment of the ligated DNA was performed using i5 and i7 (index) primers. Finally, 12 cycles of PCR were used to produce the libraries. The quality of each library was verified using Qubit dsDNA HS assay (Thermo Fisher, Waltham, United States) and Agilent 2100 Bioanalyzer. The libraries were pooled, diluted to 4 nM and sequenced using the MiSeq Reagent Kit v3 600 cycles (Ilumina, California, USA) following the manufacturer’s recommendations.

### RNA-seq analysis

Treatment with exclusively 2,4-TDA yielded lower bacterial biomass and RNA concentrations and thus, a pooled sample was sequenced, lacking replicates. Pre-alignment quality control (FASTQC), trimmed and mapping reads to the annotated genome (deposited at DDBJ/ENA/GenBank under the accession WOVH00000000) using Bowtie 2 preceded. The analysis of this data was carried out with the DESeq2 package (Bioconductor)^47^. The analysis was fundamentally based on the assessment of the whole transcriptomics profile among treatments, normalization by size factor, filtering of unexpressed genes (≤ 10 reads among all replicates and treatments), and identification of the most overexpressed genes, comparing those treatments containing 2,4-TDA to succinate. Those genes reported on Table S1 represent the top 200 most overexpressed genes in either of the treatments containing 2,4-TDA.

## Results and discussion

In 2020, Cárdenas Espinosa and colleagues suggested a degradation pathway for 2,4-TDA as the common precursor and degradation intermediate of polyurethanes in *P. capeferrum* TDA1^31,34,48^. The closest type strain species based on the genome-genome comparison (digital DNA-DNA hybridization) for the organism is *Pseudomonas capeferrum* WCS358^49^. TDA1 and WCS358 can be found in the same species cluster and differ significantly from the *P. putida* type species. The mineralization of 2,4-TDA was proposed to involve an extradiol cleavage of the aromatic compound, as well as two deamination reactions^34^. Here, further insights and proof regarding the mentioned metabolic pathway in *P. capeferrum* TDA1 are provided from transcriptomic data.

The formation of polyphenols and metabolic intermediates as a result of the bacterial activity on the substrate 2,4-TDA, posed a challenge for RNA extraction^46,50,51^. For this reason, an optimized method was established to prevent the interaction of such components with nucleic acids, and to yield RNA of higher quantity and quality than commercial kits and traditional methods^46^. Taking advantage of this new approach, total RNA extraction was conducted from samples of *P. capeferrum* TDA1 grown on 2 mM 2,4-TDA (Figure S2), 2 mM 2,4-TDA supplemented with succinate, or succinate only (as a control). These samples were then complemented with RNA-seq. The presence of the aromatic compound induces a stress response^52^ and its degradation, even if it is not the main carbon source. RNA-seq data showed no striking difference between the two 2,4-TDA-containing treatments (Figure S3). PCA clustered each treatment independently, which highlights a particular response associated to the carbon source in the media (Figure S3b). In addition to the PCA results describing a comparable response, 2,4-TDA-containing treatments shared transcriptomics characteristics showing a similar gene expression pattern.

### Transcriptional changes in TDA1 upon exposure to the PU monomer 2,4-TDA

**Figure 1:**
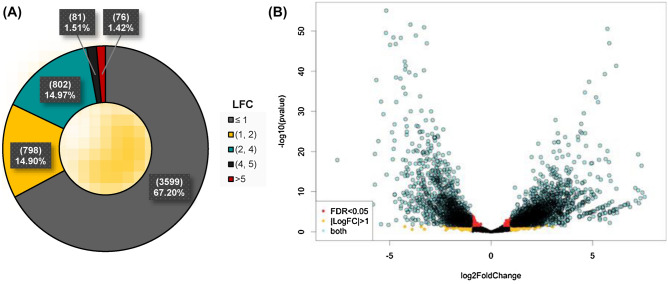
Analysis of differentially expressed genes in 2,4-TDA treated *P. capeferrum* TDA1 compared to a succinate-cultivated control. (**A**) Fractions of differentially expressed genes (DEG) by log2 Fold Change (LFC, logFC). DEGs with LFCs below 2 were mostly not considered for further analysis. (**B**) Volcano plot of the DEG in respect of their p-value (also FDR). *FDR* = *False Discovery Rate* = *p-value.* Candidates for significant DEG are characterized by a high p-value and high logFC.

Initially, the whole differential expression in *P. capeferrum* TDA1 treated with 2,4-TDA compared to succinate was analyzed. One third of the expressed genes in strain TDA1 grown on 2,4-TDA were overexpressed in comparison to the control (Fig. 1A). From those, 157 genes were expressed more than four-fold. A similar pattern was observed for downregulated genes (Fig. 1B). These results clearly denote the enormous effect that aromatic compounds such as 2,4-TDA have at the cellular transcriptional regulation, including not only their catabolism, but also their transport and defense against the inherent toxicity of these compounds, among many others which are covered subsequently^53–55^.

**Figure 2:**
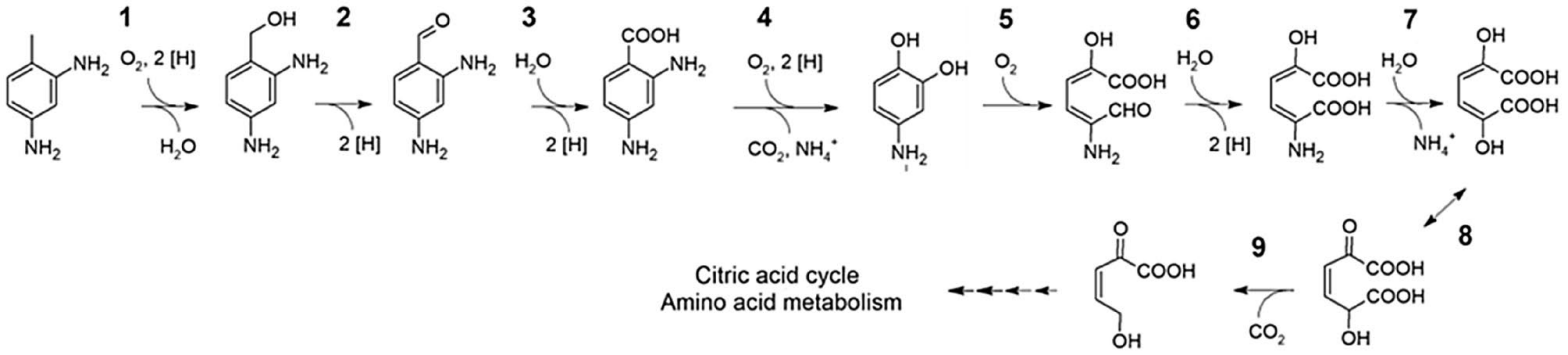
Proposed degradation pathway for 2,4-TDA in *Pseudomonas capeferrum* TDA1, via 4-aminoanthranilate (3), 4-aminocatechol (4), 4-amino-2-hydroxy-muconate semialdehyde (5) and 4-amino-2-hydroxy-muconate (6). The lower degradation pathway of 2,4-TDA needs to be elucidated in further studies.

The biochemistry of upper intracellular degradation pathway of 2,4-TDA was extensively discussed earlier^34^, and one primary aim of the present work was to validate the degradation pathway in *P. capeferrum* TDA1 and to further elucidate its key players. Several candidates of the previously suggested catabolic pathway were identified to be highly overexpressed in TDA1 cells treated with 2,4-TDA (Fig. 2, Table 1). In Table 1, a candidate list of genes involved in the catabolism of the aromatic compound is presented based on their molecular function, as well as their differential expression when compared to succinate. Interestingly, a complete operon encompassing all the members of the degradation pathway was not detected although in this subset of genes many are located in close vicinity to other genes involved in the pathway (Table 1).

**Table 1:** List of gene candidates for the intracellular degradation pathway of 2,4-TDA in *P. capeferrum* TDA1 (*see* Fig. 2).

No.	Gene	2,4-TDA v. Succ		2,4-TDA + Succ		Annotated function	Uniprot^c^
		Log2 fold change^a^	padj^b^	Log2 fold change^a^	padj^b^		
1	tsaM1_2*	3.40 ± 1.31	0.0545	3.34 ± 0.50	1.16E-10	4-Toluene sulfonate methyl-monooxygenase	GNP06_06615
	pobB_1*	3.31 ± 1.11	0.0222	2.48 ± 0.42	9.95E-09	4-Toluene sulfonate methyl-reductase subunit	GNP06_06620
2	*adhB_1*	3.40 ± 1.33	0.0586	3.38 ± 0.51	1.77E-10	Alcohol dehydrogenase (quinone)	GNP06_06700
3	*feaB_1*	3.88 ± 1.22	0.0135	3.48 ± 0.52	1.27E-10	Phenylacetaldehyde dehydrogenase	GNP06_05050
4	benA**	4.16 ± 3.16	0.0428	4.27 ± 0.63	7.61E-11	2-halobenzoate 1,2-dioxygenase large subunit	GNP06_08315
	benB**	3.71 ± 1.94	0.1751	3.55 ± 0.78	1.61E-05	2-halobenzoate 1,2-dioxygenase small subunit	GNP06_08310
	benC**	5.83 ± 3.01	0.1700	6.80 ± 1.21	7.00E-08	Benzoate 1,2-dioxygenase electron transfer component	GNP06_08305
5	hpaD***	2.32 ± 1.05	0.1117	1.97 ± 0.35	4.12E-08	Ring-cleaving dioxygenase	GNP06_05110
6	hpaE***	2.82 ± 1.52	0.1927	3.31 ± 0.56	1.32E-08	5-carboxymethyl-2-hydroxymuconic semialdehyde dehydrogenase	GNP06_05115
7	*GNP06_05035*	6.02 ± 2.85	0.1295	7.23 ± 1.20	8.51E-09	Aminomuconate deaminase	GNP06_05035
8 + 9	hpcE_1***	2.76 ± 1.73	0.2721	3.30 ± 0.59	7.69E-08	Homoprotocatechuate catabolism bifunctional isomerase/decarboxylase	GNP06_05120

*/**/***Adjacent genes (to each other).

^a^log2 Fold Change ± standard error (LFC).

^b^p-value adjusted (versus control, i.e., succinate).

^c^Uniprot refers to the accession number of the TDA1 protein in this database.

Briefly, the degradation of 2,4-TDA is most probably initiated by adjacent genes *tsaM_1* (candidate gene GNP06_06615) and *pobB_1* (gene GNP06_06620), which could encode the formation of a primary alcohol, given the similarity of 2,4-TDA to toluene 4-sulfonate. 4-aminoanthranilate (2,4-aminobenzoate) could be formed as a result of the function of an alcohol dehydrogenase and subsequently, an aldehyde dehydrogenase. *adhB_1* and *feaB_1* (candidate genes GNP06_06700 and GNP06_05050, respectively) could foster these reactions, given their transcriptional overexpression pattern and their ability to act on a broad range of aromatic substrates^56–58^. Nonetheless, other aldehyde dehydrogenase genes, such as *paoABC* homologs (accession P77324) or *aldH* (candidate gene GNP06_06665) were also significantly overexpressed. Since the substrate specificity of these enzymes is rather broad, the possibility that other proteins could also fulfill this function cannot be excluded^59,60^. Subsequently, a first deamination could be promoted nonspecifically by the action of benzoate 1,2-dioxygenase activity of the highly overexpressed cluster *benABC* (genes GNP06_08305, GNP06_08310 and GNP06_08315), yielding 4-aminocatechol.

It was also proposed that the intracellular degradation pathway of 2,4-TDA encompasses an extradiol ring cleavage of 4-aminocatechol yielding 4-amino-2-hydroxy-muconate semialdehyde; which is most possibly coded by dioxygenase *hpaD* (GNP06_05110). Nonetheless, the dioxygenase *pcaH*, which promotes an *ortho-*cleavage of the 3,4-dihydroxybenzoate aromatic ring, was also notably overexpressed in the presence of 2,4-TDA (*data not shown*). Encoded by the adjacent gene *hpaE* (GNP06_05115), 5-carboxymethyl-2-hydroxymuconic semialdehyde dehydrogenase could yield 4-amino-2-hydroxy-muconate.

Finally, the uncharacterized gene GNP06_05035*,* which encodes a putative aminomuconate deaminase, could enable the second deamination step. This gene holds many homologues in the *Pseudomonas* genus, which have been annotated as RidA family proteins, as well as enamine/imine deaminases. Nonetheless, despite its significant upregulation, it might also be possible that the second deamination step could take place in the lower degradation pathway, as part of the amino acid metabolism, namely given the overexpression of other deaminases, such as the 2-iminobutanoate/2-iminopropanoate deaminase GNP06_17020, among other transaminases and aminotransferases. HpcE_1 (gene GNP06_05120), whose gene is adjacent to *hpaD* and *hpaE*, could further tautomerize and carboxylate the resulting intermediate 2,5-dihydroxy-muconate^34^. The lower degradation pathway of 2,4-TDA needs to be elucidated in further studies.

### Induction of general stress response and biofilm formation

To tackle the inherent toxic effects of 2,4-TDA, *P. capeferrum* TDA1 has evolved complicated and sophisticated mechanisms to defend itself against the toxicity of suchlike compounds, yet still being able to use 2,4-TDA as a carbon, nitrogen and energy source^34^. Several genes involved in the general stress response of the cell against a wide range of abiotic environmental stresses showed a significant overexpression in the transcriptomic data set. For instance, genes implicated in the transport, protein misfolding protection and regulation against metal ions stress presented an increased transcriptional expression (Table S1) when 2,4-TDA was present. In the last decades, several reports showcased a similar general stress response in the toluene-tolerant *P. putida* DOT-T1E and *P. putida* S12 in the presence of toluene^61–64^.

Furthermore, many genes involved in biofilm formation and bacterial motility were highly expressed in 2,4-TDA treatments when compared to succinate culture. Genes such as diguanylate cyclase GNP06_08145, glutathione transport system permeases *gsiD* and *gsiC* or the *alg* operon were also present in the most overexpressed genes in 2,4-TDA-containing treatments (Table S1). The *alg* operon, formed by *algA, algE, algF*, among many others, is involved in the production and transport of alginate, an exopolysaccharide found in the biofilm matrix, especially produced by the genus *Pseudomonas*^65^. Tripeptide glutathione may impede the formation of bacterial biofilms^66^. Hence the upregulation of glutathione transport, concurred with its non-differentially expressed biosynthetic pathway in the presence of 2,4-TDA (*data not shown*).

It was previously documented that the tricarboxylic (TCA) cycle components were upregulated in the presence of hydrocarbon solvents, in order to modulate the NAD(P)H concentration^63^. It was discussed, that such upregulation could enable the bacteria to cope with the energetic potential loss related to other defense mechanisms, such as solvent efflux pumps and maintenance of redox balance^67^. However, TCA cycle upregulation was not observed in our strain TDA1, similarly to the results obtained in *P. putida* DOT-T1E^68^. Given that DOT-T1E could tolerate higher concentrations and degrade toluene, the authors concluded that the induction of the toluene degradation pathway eclipsed the need to upregulate the TCA cycle^68^. Thus, it is likely that the capability to degrade 2,4-TDA and use this compound as energy, carbon and nitrogen sources prevents the induction of other central metabolism pathways, such as the TCA cycle.

Finally, another defense mechanism used by bacteria to endure in high concentrations of toxic aromatic compounds is actively pumping them out into the extracellular space^54,61^. From a vast subset of enzymes involved in this defense mechanism, efflux pumps, multidrug resistance proteins, RND transporters, as well as specific porins were largely overexpressed in the presence of 2,4-TDA (Table S1). Among these, several proteins of the *ttg (toluene tolerance genes)* efflux systems were found, such as *ttgI* and *ttgC* (Table S1). Nonetheless, the annotated *ttgRABC operon* was not differentially expressed (*data not shown*), which correlates with the downregulation of the TCA cycle: *P. capeferrum* TDA1 endures the inherent toxicity of 2,4-TDA by actively degrading it.

### Strategies and enzymes for the extracellular cleavage of PU compounds in P. capeferrum TDA1

Pseudomonads show a promising metabolic potential and high adaptability to a broad range of environmental stresses. Besides, *P. capeferrum* TDA1 can not only tolerate and use a PU monomer as carbon, energy and nitrogen source, but was also observed to degrade a PU oligomer^34^. These features make the strain an interesting candidate for biological recycling. For this sort of applications, the extracellular strategies of the strain to unlock PU compounds are of eminent importance and thus were assessed further in this work.

### P. capeferrum TDA1 possesses a membrane-bound esterase activity

**Figure 3:**
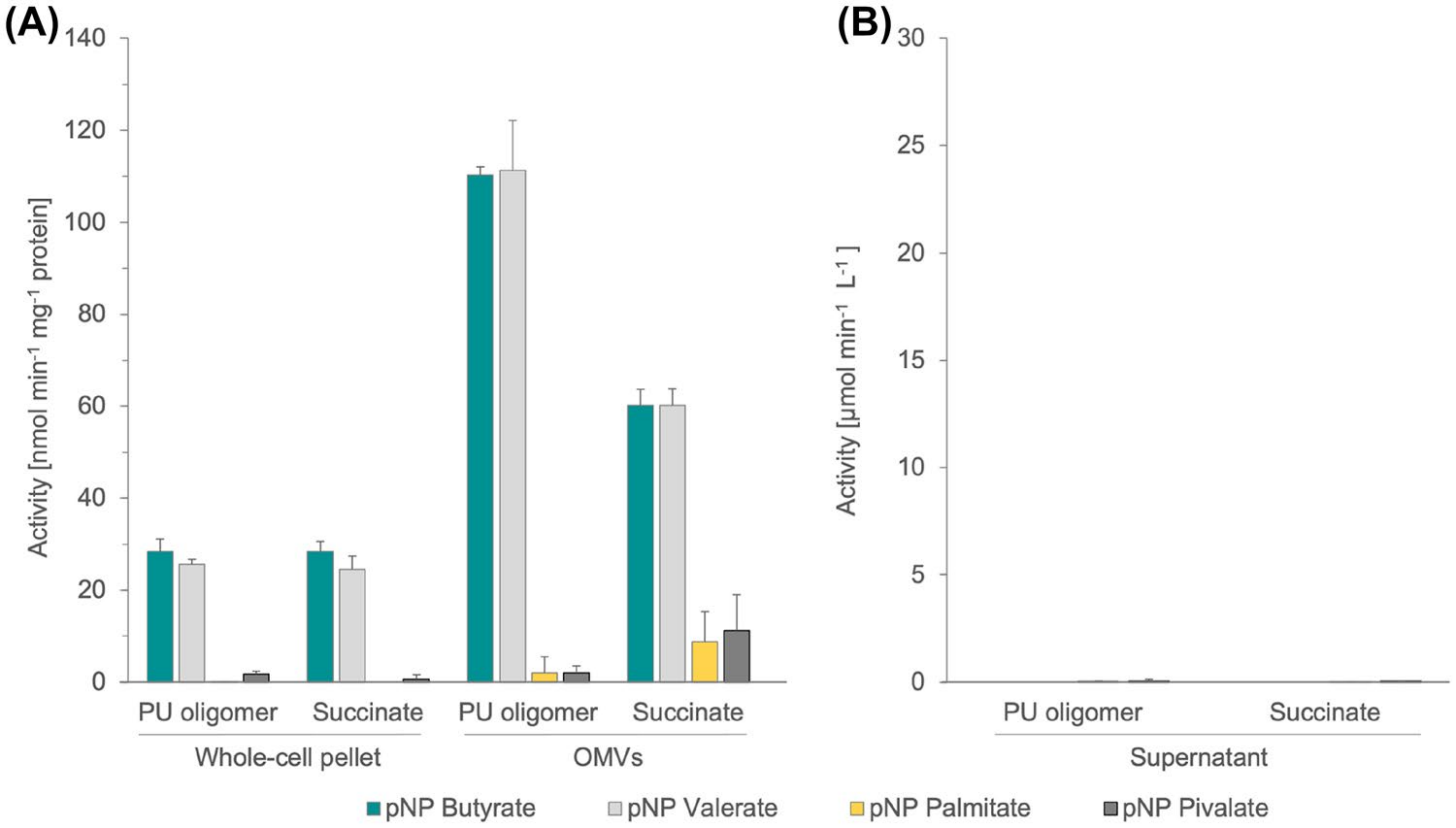
Conversion of *p*-nitrophenyl esters to *p-*nitrophenol by different fractions of *P. capeferrum* TDA1 grown in PU oligomer, or succinate (control). (**A**) Esterase/Lipase activity on whole-cell pellets and outer-membrane vesicles (OMVs) (per mg protein) and (**B**) supernatants (per volume) of *P. capeferrum* TDA1 were assessed. OMV samples were subjected to a protein quantification via BRADFORD. Standard deviations and legend are given.

PU extracellular degradation occurs via the action of extracellular and membrane-bound hydrolases^13^. Even though *P. capeferrum* TDA1 does not contain any known extracellular polyurethanase homolog (*e.g.* PueA from *P. protegens* Pf-5; *data not shown*), esterase activity has been previously identified to act on polyester-based polyurethane^48,69,70^. Thus, the ability of *P. capeferrum* TDA1 to cleave different *p-*nitrophenyl (*p*NP) esters was assayed^41^. Cultures of TDA1 were grown in PU oligomer or succinate (control) and their supernatant, whole-cell pellets and outer membrane vesicles (OMVs) were isolated and investigated separately. As it can be noticed in Fig. 3A, esterase activity was only detected in whole-cell pellets and OMVs, but not in the supernatant (Fig. 3B), regardless of the carbon source applied. Moreover, the cultivation of TDA1 with the PU oligomer did not induce a significant rise in the esterase activity for the *p*NP substrates analyzed compared to the control. Hence, the obtained results suggest the presence of an outer membrane-bound esterase activity in *P. capeferrum* TDA1. Moreover, the activity was two- and four-fold increased for isolated OMVs compared to the cell pellets.

OMV release in TDA1 was shown to increase significantly in PU oligomer compared to succinate, with relative OMV yields of 0.28 ± 0.05% and 0.09 ± 0.01%, respectively. This three-fold increase shows that the release of OMV is part of the adaptive mechanisms of bacteria to stressful environmental conditions such as the presence of toxic PU compounds, as previously discussed for other organic contaminants^71–73^. In addition, OMV may also be used as vehicle to transport the necessary enzyme through extracellular space to unlock new carbon sources like the PU oligomer.

### Enzyme candidates identified in OMV fractions

**Figure 4:**
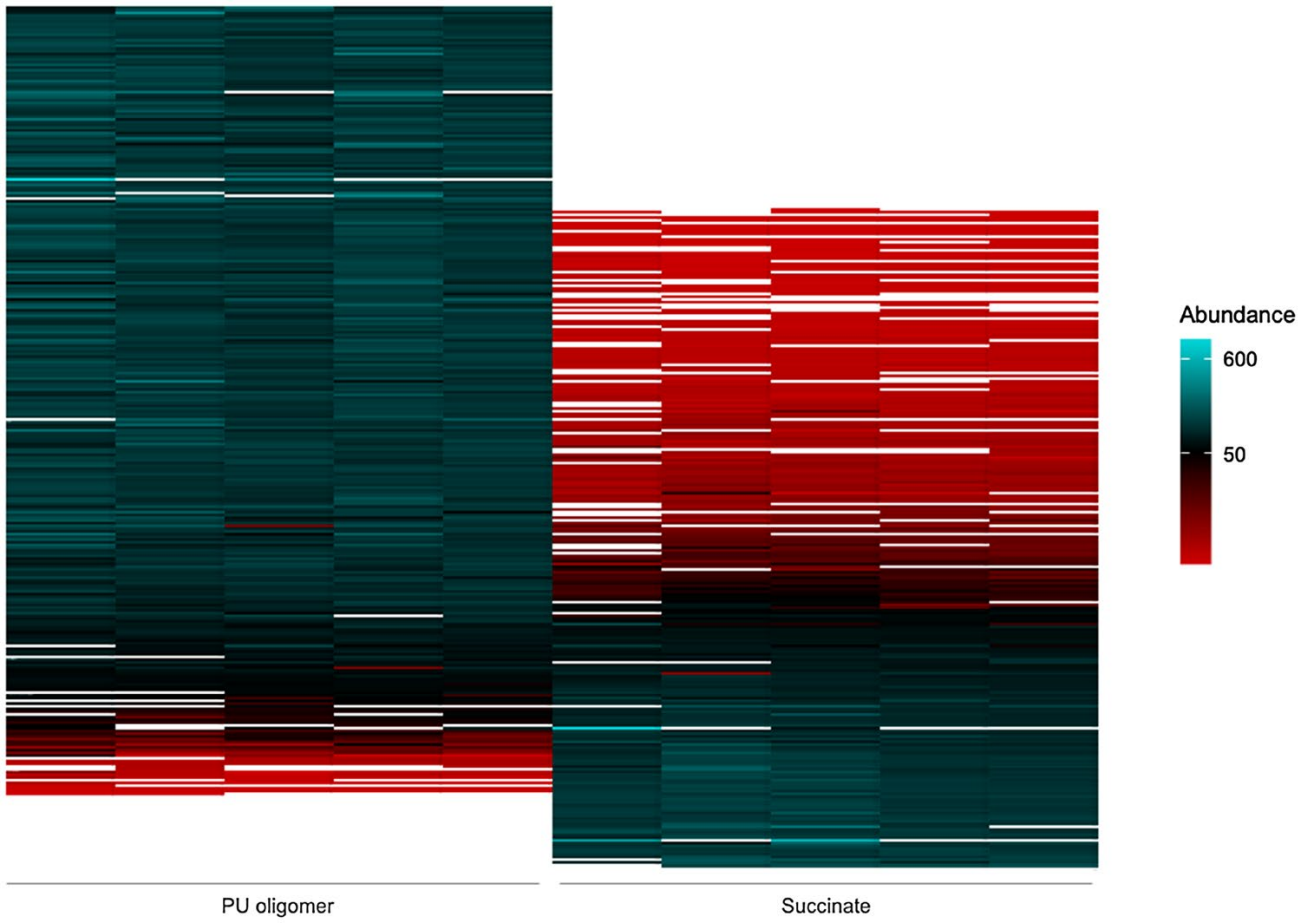
Heatmap of extracellular proteins detected by mass spectrometry on OMV fractions of TDA1 in PU oligomer or succinate showing the difference of the samples. Abundance for each of the five replicates of each treatment is given as a color scale, being *white* non detected enzymes.

In order to identify the enzymes involved in the cleavage of *p-*nitrophenyl esters and potentially oligomeric PU, OMVs of cells grown on PU oligomer or succinate were analyzed by LC–MS. 318 proteins which are localized –or predicted to be localized– in the periplasm, flagellum, outer membrane or extracellularly were identified. Among those, 95 proteins have not been characterized yet or they possessed domains of unknown function (DUF). Yet, a substantial difference in TDA1’s OMV-exoproteome among treatments was perceived (Fig. 4, S4), which suggests that each carbon source induces a distinct and characteristic subset of extracellular proteins and concatenated reactions. However, the esterase activity of both subsets seem to be in the same order of magnitude regardless of the carbon source. Similar results showed that nanopod/OMV formation was induced by growth of *Delftia acidovorans* Cs1-4 on phenanthrene^21^. Furthermore, the study suggested the contribution of extracellular structures as elements supporting metabolic biodegradation processes.

**Figure 5:**
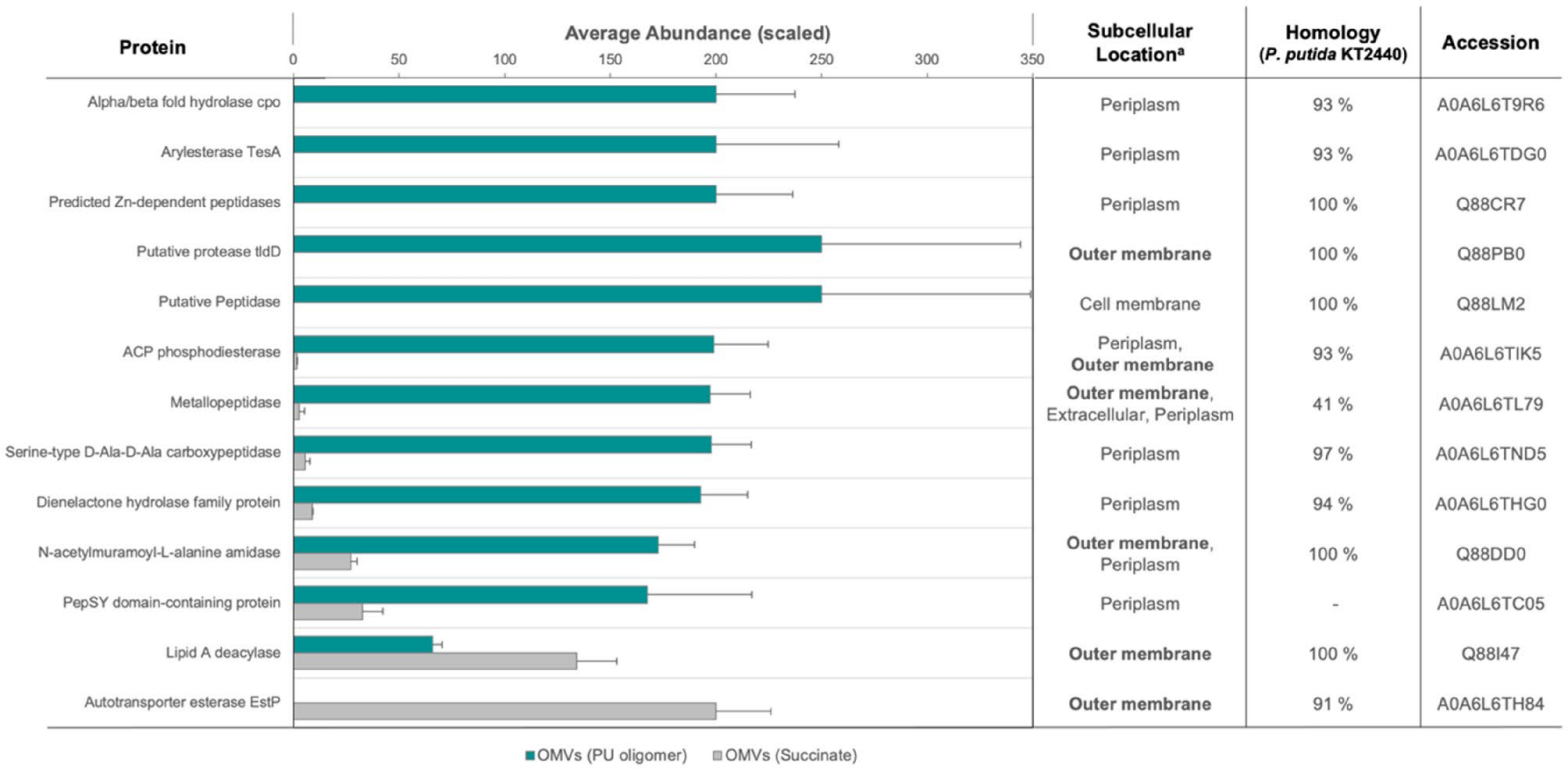
Candidate list from mass spectrometry of OMV samples in both PU oligomer and succinate. Candidates were selected due to their abundance in the OMV samples, their subcellular location, and their homology to proteins in *P. putida* KT22440. ^a^ *In the case that subcellular location was not annotated for the respective protein, prediction *via* CELLO v.2.5.* The proteomes of OMVs from cells grown on succinate and OMVs from cells on the PU oligomer clearly differ from each other and esterases were detected in both conditions.

A subset of the hydrolases detected in OMV fractions with annotations that suggest a putative cleaving-function of ester bonds or amid-type bonds was included in Fig. 5. Interestingly, several candidates capable of executing the esterase cleavage were identified. The esterases TesA and EstP were identified, whose homologs have been previously spotted in OMVs of different *Pseudomonas spp.*^74,75^ and linked in *P. aeruginosa* and *P. putida* to the cleavage of *p-*nitrophenyl esters of short acyl chains^76,77^. Notably, these characterized esterases were exclusively detected in OMV of the PU culture (TesA) *or* in the OMV fraction of the succinate culture (EstP), which could indicate a regulation of each esterase in a substrate-specific manner. Nevertheless, such substrate-specific effect on the exo-esterase expression has not yet been investigated and further research will be needed to prove these hypotheses.

Other proteins such as, amidases, peptidases, proteases, or Lipid A deacylase could also contribute to the cleavage of *p-*nitrophenyl esters of short acyl chain, although only one or a few of them may be dominant. The mentioned hydrolases and thus the extracellular degradation pathway could be conserved among *Pseudomonas* spp. and potentially Proteobacteria (Fig. 5).

### A possible strategy to enhance the degradation of a polyurethane oligomer by the action of hydrolases present in OMVs in P. capeferrum TDA1

**Figure 6:**
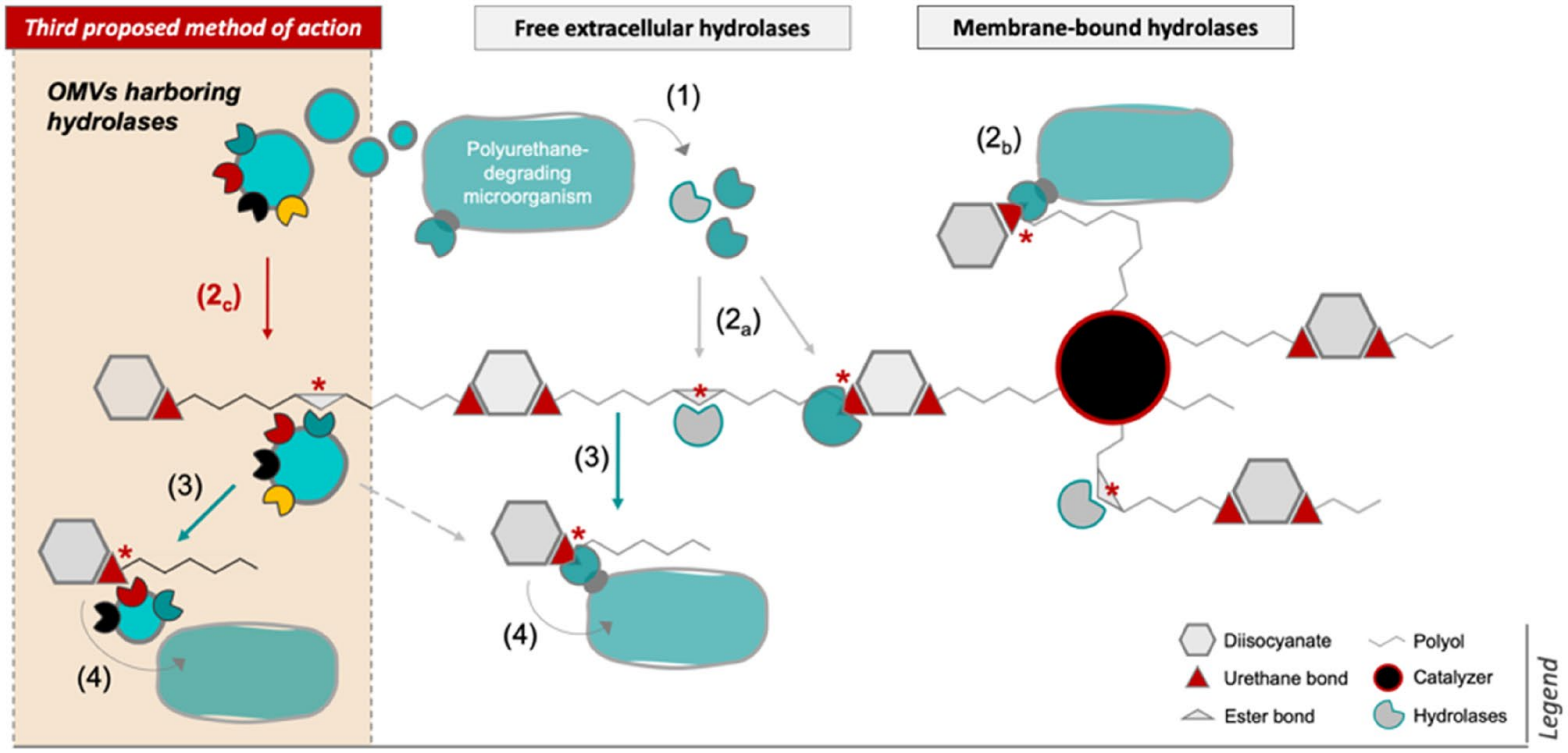
Model for extracellular degradation of polyester-based polyurethane via OMV function by *P. capeferrum* TDA1. A new mechanism to biodegrade extracellular polyurethane through the release of outer membrane vesicles (OMVs) by the bacterial cell is suggested. These OMVs could harbor outer membrane-bound or periplasmic hydrolases to promote degradation (2_c_). In the case of the latter, their function would occur through the lysis of the OMVs (*not depicted*). Procedure: (1) Degradative microorganisms express free and membrane-bound hydrolytic enzymes, and release OMVs. Enzymatic degradation (*) occurs through (2_a_) extracellular enzymes and (2_b_) outer membrane-bound hydrolases, which can also be present in (2_c_) OMVs. As a result, (3) smaller PU fragments may be released and further cleaved, (4) which can be finally transported in the cell and mineralized.

Microbial plastic degradation has been extensively studied in the last decades, yet a relation with OMVs harboring hydrolytic enzymes has never been presented. Nonetheless, OMVs have been identified recently to induce lignin-derived aromatic compound degradation in *P. putida* KT2440^20^. Interestingly, the authors suggested a model through which hydrolytic periplasmic enzymes in secreted vesicles could also promote degradation via OMV lysis^20^. Thus, macromolecular degradation may occur through the action of both periplasmic and membrane-bound hydrolases harbored in OMVs. According to the results presented in this work, OMVs can be considered as a supporting mechanism for biodegradation (Fig. 6). This proposed method would function alongside free extracellular enzymes and membrane-bound hydrolases, as is widely recognized^3,13^. The uptake of catabolic products would occur subsequently by the bacterial cell, which would promote the mineralization of the compound. Moreover, we surmise that such model could be conserved among *Pseudomonas* spp. and potentially *Proteobacteria,* given that OMV release is an ubiquitous defense mechanism among Gram-negative bacteria^72,78,79^.

## Conclusion and future perspectives

Biodegradation of aromatic substrates by *Pseudomonas* contributes significantly to pollutant removal in various ecosystems. Understanding of the biochemical pathways may provide vital information for enhancing catabolic efficiency. The results presented in this work demonstrate that *P. capeferrum* TDA1 degrades PU monomers efficiently. Thus, TDA1 could be preferentially employed in a two-step degradation process, in which enzymatic catalysis of the macromolecular polymer initially yielded plastic monomers. Furthermore, *P. capeferrum* TDA1 could use these monomers to synthesize novel value-added products in a new circular plastic economy, as recently studied^80^.

OMVs and external cell structures have been proven important in numerous microbial activities including biodegradation^20,21^. It can be expected that free and membrane-bound hydrolases alongside play a role in the extracellular degradation of PU monomers and oligomers, as described in our proposed model (Fig. 6).

## Supplementary Information

Supplementary Information.
