# Supplementary material for: Extracellular degradation of a polyurethane oligomer involving outer membrane vesicles and further insights on the degradation of 2,4-diaminotoluene in Pseudomonas capeferrum TDA1
Source: Sci Rep. 2022 Feb 17;12:2666. doi: 10.1038/s41598-022-06558-0 (PMC8854710; doi:10.1038/s41598-022-06558-0)
Supplement: Supplementary file 1 — Supplementary Information. [file 41598_2022_6558_MOESM1_ESM.docx]

**Extracellular degradation of a polyurethane oligomer involving outer membrane vesicles and further insights on the degradation of 2,4-diaminotoluene in *Pseudomonas capeferrum* TDA1**

Òscar Puiggené^1,6,^°, María José Cárdenas Espinosa^1,^°, Dietmar Schlosser^2^, Stephan Thies^3^, Nico Jehmlich^4^, Uwe Kappelmeyer^1^, Stephan Schreiber^4^, Daniel Wibberg^5^, Joern Kalinowski^5^, Hauke Harms^2^, Hermann J. Heipieper^1^*, Christian Eberlein^1^

*^1^Department of Environmental Biotechnology, Helmholtz Centre for Environmental Research - UFZ, Leipzig, Germany.*

*^2^Department of Environmental Microbiology, Helmholtz Centre for Environmental Research - UFZ, Leipzig, Germany.*

*^3^Heinrich Heine University Dusseldorf, Institute of Molecular Enzyme Technology, Jülich, Germany.*

*^4^Department Molecular Systems Biology, Helmholtz Centre for Environmental Research - UFZ, Leipzig, Germany.*

*^5^Microbial Genomics and Biotechnology, Center for Biotechnology (CeBiTec), Bielefeld, Germany*

*^6^The Novo Nordisk Foundation Center for Biosustainability, Technical University of Denmark, 2800 Kgs, Lyngby, Denmark*

*°These authors contributed equally.*

**Corresponding author’s phone and e-mail: +493412351694, hermann.heipieper@ufz.de*

Supplementary Information

**Figure S1. Growth kinetics of *P. capeferrum* TDA1 grown on PU oligomer.**

**Figure S2. Growth kinetics of *P. capeferrum* TDA1 grown on 2,4-TDA.**


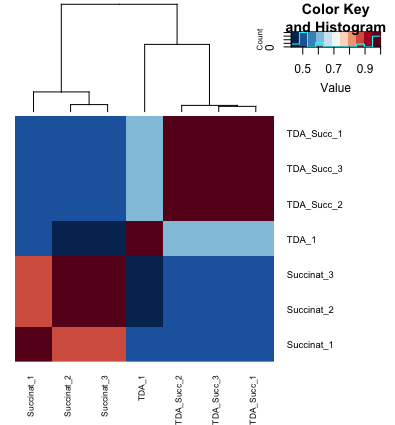


**(B)**

**(A)**


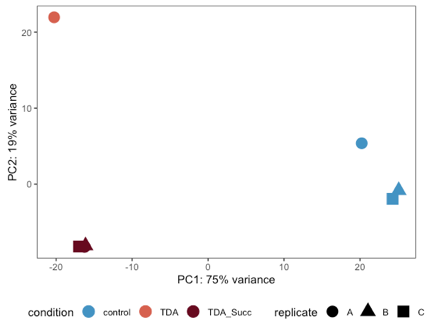


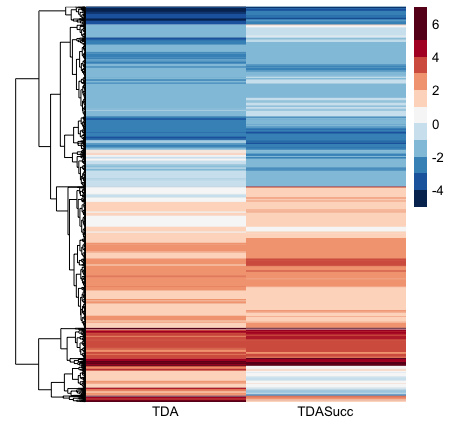


**(C)**

Figure S3. Comparison of treatments 2,4-TDA (*TDA*) and 2,4-TDA supplemented with succinate (*TDASucc*). (A) Pair-wise correlation heatmap (using spearman’s correlation method) per replicate. (B) PCA of normalized RNAseq read counts. *(A, B) also include succinate, control samples*. C) Heatmap of differentially expressed genes of each treatment when compared to control samples.


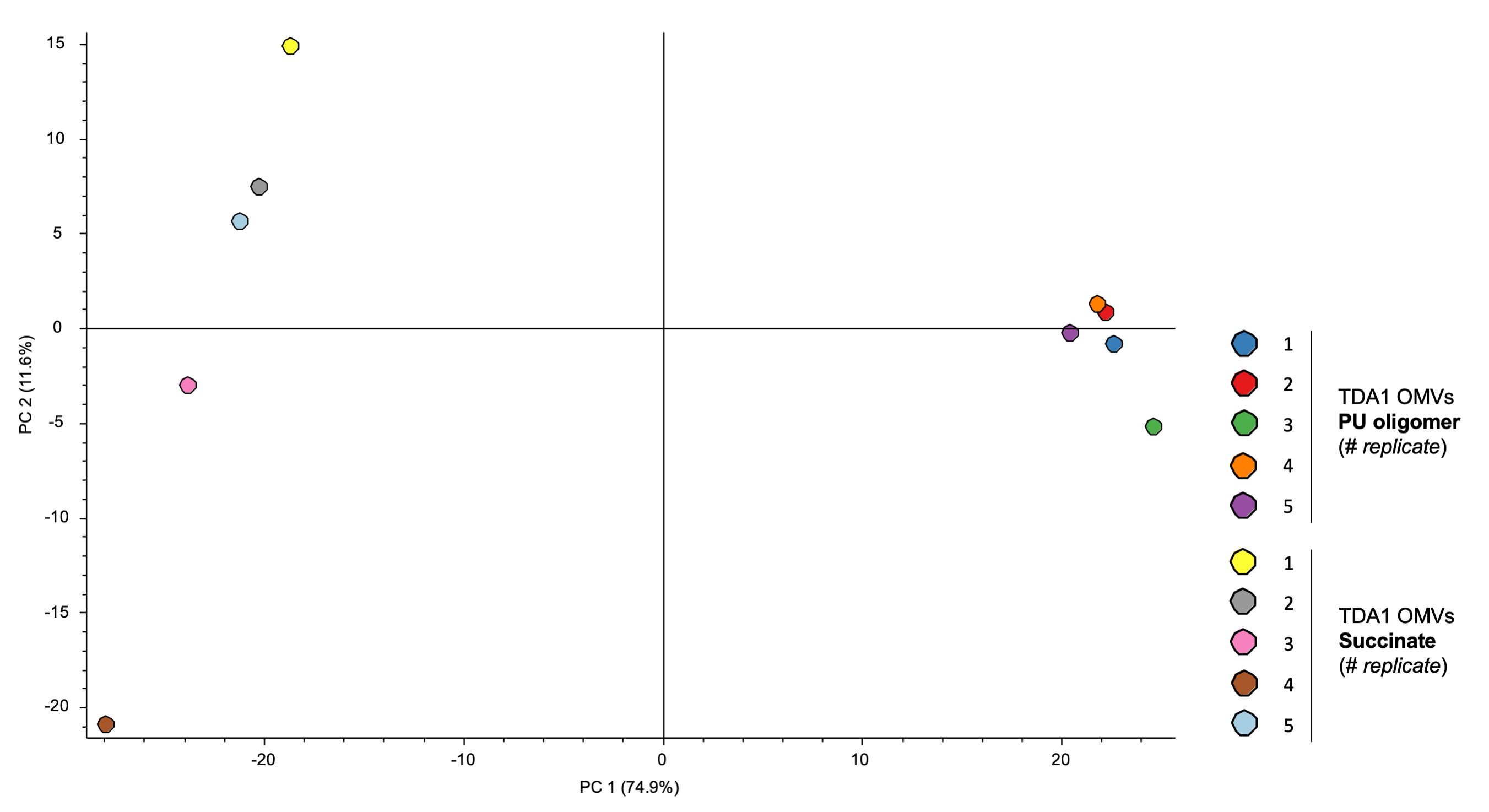
Figure S4. PCA Analysis of *P. capeferrum* TDA1 exoproteome of OMVs grown in PU oligomer or succinate. This analysis, though, also includes cytosolic proteins in low concentrations yet considered as contamination of the OMV sample. Legend is given.

Table S1. List of genes with a high overexpression pattern grouped by function retrieved from the RNA-Seq data of *P. capeferrum* TDA1 cells grown on 2,4-TDA or 2,4-TDA supplemented with succinate (Succ) compared to the control (succinate). p-value adjusted (padj), as well as the presented fold change values of the correlation between treatments 2,4-TDA and succinate displayed considerably higher numbers compared to the treatment 2,4-TDA supplemented with succinate due to the existence of a single 2,4-TDA replicate, which is heavily penalized by the normalization algorithm. Nonetheless, such replicate was composed by a pool of cultures – for biomass reasons.

| ***gene*** | **2,4-TDA v. Succ** | | **2,4-TDA + Succ v. Succ** | | **Gene Function** | **Uniprot^c^ (homology)** |
| --- | --- | --- | --- | --- | --- | --- |
|  | **LFC^a^** | **padj^b^** | **LFC** | **padj** |  |  |
| **Genes involved in aromatic compound degradation** | | | | | | |
| *paaE* | 7,1096 | 1,01E-02 | 7,1870 | 1,28E-08 | 1,2-phenylacetyl-CoA epoxidase, subunit E | P76081 |
| *ahpF* | 6,1665 | 5,04E-11 | 1,5914 | 2,81E-02 | Alkyl hydroperoxide reductase subunit | P35340 |
| *Hgd* | 6,1336 | 4,31E-13 | 1,8883 | 9,24E-06 | 2-(hydroxymethyl)glutarate dehydrogenase | Q0QLF5 |
| *paaI_2* | 6,1095 | 1,13E-01 | 5,9584 | 2,98E-06 | Acyl-coenzyme A thioesterase PaaI | P76084 |
| *paaB* | 6,0221 | 1,30E-01 | 5,8149 | 7,51E-06 | 1,2-phenylacetyl-CoA epoxidase, subunit B | P76078 |
| *gudD* | 5,9329 | 7,18E-03 | 6,6207 | 8,94E-10 | Glucarate dehydratase | P42206 |
| *hcaD* | 5,8580 | 1,38E-04 | 4,7354 | 2,04E-09 | 3-phenylpropionate/cinnamic acid dioxygenase ferredoxin-NAD(+) reductase component | J7R3Y9 |
| *mdlC* | 5,3875 | 2,73E-08 | 2,2273 | 2,52E-04 | Benzoylformate decarboxylase | P20906 |
| *aroH* | 5,3535 | 1,76E-13 | -0,4249 | 3,52E-01 | Phospho-2-dehydro-3-deoxyheptonate aldolase | P80574 |
| *paaH* | 5,2235 | 3,94E-01 | 4,3773 | 9,89E-08 | 3-hydroxyadipyl-CoA dehydrogenase | P76083 |
| *glaH* | 5,0320 | 8,07E-03 | 4,4507 | 1,66E-08 | Glutarate 2-hydroxylase | P76621 |
| *GNP06_17000* | 5,0204 | 8,04E-03 | 4,4466 | 2,22E-08 | 4-sulfomuconolactone hydrolase | A6XIG7 |
| *cntA* | 4,7977 | 1,89E-02 | 4,7430 | 2,35E-09 | Carnitine monooxygenase oxygenase subunit | D0C9N6 |
| *paaA* | 4,4554 | 4,52E-02 | 4,4715 | 1,32E-08 | 1,2-phenylacetyl-CoA epoxidase, subunit A | P76077 |
| *gudP_1* | 4,3711 | 2,47E-02 | 4,6211 | 1,26E-12 | putative glucarate transporter | Q46916 |
| *hipO* | 4,3548 | 6,02E-02 | 4,3707 | 7,33E-07 | Hippurate hydrolase | P45493 |
| *aroE_1* | 3,5101 | 2,32E-01 | 4,0876 | 5,54E-07 | Shikimate dehydrogenase (NADP(+)) | Q8Y9N5 |
| *quiA_1* | 3,0324 | 4,49E-02 | 4,0234 | 3,01E-17 | Quinate/shikimate dehydrogenase (quinone) | Q59086 |
| *aroQ* | 2,9849 | 3,81E-01 | 3,9177 | 1,52E-06 | 3-dehydroquinate dehydratase | P43877 |
| **Genes regulated by general stress response involved in metal ions-related functions** | | | | | | |
| *czcC_1* | 7,3440 | 4,21E-03 | 7,4661 | 2,91E-09 | Cobalt-zinc-cadmium resistance protein CzcC | P13509 |
| *zupT_2* | 6,1680 | 1,90E-17 | 1,8562 | 1,79E-08 | Zinc Transporter ZupT | – |
| *yciC* | 5,8081 | 1,07E-13 | 2,5025 | 5,75E-13 | Putative metal chaperone YciC | P94400 |
| *cueR_1* | 4,8645 | 1,10E-01 | 4,9975 | 6,75E-06 | HTH-type transcriptional regulator CueR | Q93CH6 |
| **Genes involved in the pump out of aromatic compounds** | | | | | | |
| *bepF_1** | 7,2169 | 4,40E-09 | 3,6703 | 5,18E-06 | Efflux pump periplasmic linker BepF | Q8FWV8 |
| *bepF_2* | 7,1753 | 1,17E-01 | 2,9350 | 1,30E-02 | Efflux pump periplasmic linker BepF | Q8FWV8 |
| *mdtA_4* | 6,8294 | 2,42E-02 | *not signif. expressed ^d^* | | Multidrug resistance protein MdtA | P76397 |
| *mdtC_3* | 6,1920 | 9,86E-02 | *not signif. expressed ^d^* | | Multidrug resistance protein MdtC | – |
| *bepE_1* | 5,7354 | 1,41E-02 | 3,7828 | 8,47E-04 | Efflux pump membrane transporter BepE | Q8G2M6 |
| *oqxB17* | 5,7895 | 4,51E-06 | 3,6505 | 2,92E-08 | multidrug efflux RND transporter permease subunit | – |
| *ttgI_1* | 5,3507 | 2,13E-03 | 2,9696 | 3,56E-04 | Toluene efflux pump outer membrane protein TtgI | Q93PU3 |
| *oqxB7** | 4,6525 | 1,89E-05 | 2,5363 | 1,08E-08 | multidrug efflux RND transporter permease subunit | A0A5E7AVI6 |
| *ttgC_3** | 3,8718 | 3,53E-02 | 0,7146 | 5,01E-01 | putative efflux pump outer membrane protein TtgC | Q88N32 |
| *aaeB_3* | 3,6700 | 1,90E-02 | 4,3776 | 1,15E-20 | p-hydroxybenzoic acid efflux pump subunit AaeB | – |
| *ttgI_4* | 3,6487 | 8,09E-02 | 3,7932 | 7,72E-11 | Toluene efflux pump outer membrane protein TtgI | Q93PU3 |
| **Genes involved in Biofilm induction and production** | | | | | | |
| *GNP06_08145* | 4,1307 | 2,33E-02 | 4,4085 | 8,13E-15 | diguanylate cyclase | A0A6L6TCN3 |
| *algF* | 5,1916 | 5,59E-02 | 5,5937 | 2,84E-07 | Alginate biosynthesis protein AlgF | Q06062 |
| *algE* | 4,8917 | 1,33E-02 | 5,4815 | 1,44E-12 | Alginate production protein AlgE | P18895 |
| *algJ* | 3,9323 | 6,08E-01 | 4,7296 | 2,47E-12 | putative alginate O-acetylase AlgJ | Q88ND3 |
| *algX* | 3,6863 | 5,37E-02 | 4,1352 | 2,13E-15 | Alginate biosynthesis protein AlgX | Q51372 |
| *algA* | 3,1104 | 7,20E-02 | 3,7643 | 3,38E-17 | Alginate biosynthesis protein AlgA | P07874 |
| *gsiC* | 5,6091 | 2,03E-02 | 5,3806 | 8,85E-07 | Glutathione transport system permease protein GsiC | P75798 |
| *gsiD* | 4,5606 | 3,54E-02 | 4,2416 | 1,06E-07 | Glutathione transport system permease protein GsiD | P75799 |
| *gloB_1* | 3,7321 | 9,31E-03 | 2,7045 | 7,68E-09 | Hydroxyacylglutathione hydrolase | – |
| **Enzymes with deaminase, transaminase and aminotransferase activity** | | | | | | |
| *phnW* | 5,2254 | 2,03E-04 | 4,0055 | 2,29E-10 | 2-aminoethylphosphonate-pyruvate transaminase | Q9I434 |
| *kce_1* | 4,6059 | 5,32E-01 | 4,7619 | 1,67E-11 | 3-keto-5-aminohexanoate cleavage enzyme | Q8RHX2 |
| *GNP06_17020* | 4,3980 | 2,08E-01 | 4,5934 | 3,45E-05 | 2-iminobutanoate/2-iminopropanoate deaminase | – |
| *argD_1* | 4,1445 | 1,37E-01 | 5,2993 | 1,26E-11 | Acetylornithine aminotransferase | Q9X2A5 |
| *MPHCKDGE_*  *04608* | 3,7016 | 3,75E-01 | 6,0630 | 2,42E-08 | Aminotransferase · Pyridoxal phosphate-dependent transferase, major domain | – |

*^a^LFC: log2 Fold Change.*

*^b^padj: p-value adjusted when compared to the control.*

*^c^Uniprot accession number of the closest homolog annotated and characterized in the database (if given).*

*^d^Genes which were not significantly expressed (≤ 10 counts considering all replicates and treatments).*

**Genes clustered together, possibly as a part of the same operon.*
